# A Research Roadmap: Connected Health as an Enabler of Cancer Patient Support

**DOI:** 10.2196/14360

**Published:** 2019-10-29

**Authors:** Gabriel Ruiz Signorelli, Fedor Lehocki, Matilde Mora Fernández, Gillian O'Neill, Dominic O'Connor, Louise Brennan, Francisco Monteiro-Guerra, Alejandro Rivero-Rodriguez, Santiago Hors-Fraile, Juan Munoz-Penas, Mercè Bonjorn Dalmau, Jorge Mota, Ricardo B Oliveira, Bela Mrinakova, Silvia Putekova, Naiara Muro, Francisco Zambrana, Juan M Garcia-Gomez

**Affiliations:** 1 Oncoavanze Seville Spain; 2 Sport & Society Research Group Faculty of Educational Sciences University of Seville Seville Spain; 3 Insight Centre for Data Analytics O’Brien Centre for Science University College Dublin, Belfield Campus Dublin Ireland; 4 Slovak University of Technology in Bratislava Bratislava Slovakia; 5 National Centre of Telemedicine Services Bratislava Slovakia; 6 Beacon Hospital Dublin Ireland; 7 Salumedia Tecnologías Seville Spain; 8 Maastricht University Maastricht Netherlands; 9 Architecture and Computer Technology Department University of Seville Seville Spain; 10 University of Southern Denmark Kolding Denmark; 11 Research Centre in Physical Activity, Health and Leisure Faculty of Sport University of Porto Porto Portugal; 12 Laboratory of Active Living Institute of Physical Education and Sports University of Rio de Janeiro State Rio de Janeiro Brazil; 13 First Department of Oncology Comenius University Bratislava Slovakia; 14 Faculty of Health Care and Social Work University of Trnava Trnava Slovakia; 15 Laboratoire d'informatique médicale et d'ingénierie des connaissances en e-Santé Sorbonne Universités Paris France; 16 eHealth and Biomedical Applications Vicomtech Donostia-San Sebastian Spain; 17 Biodonostia Donostia-San Sebastián Spain; 18 Department of Oncology Infanta Sofia University Hospital Madrid Spain; 19 Biomedical Data Science Lab The Institute of Information and Communication Technologies Universitat Politecnica de Valencia Valencia Spain

**Keywords:** cancer, Connected Health, mHealth, eHealth, mental health, physical activity, rehabilitation, wearable, Internet of Things, quality of life

## Abstract

The evidence that quality of life is a positive variable for the survival of cancer patients has prompted the interest of the health and pharmaceutical industry in considering that variable as a final clinical outcome. Sustained improvements in cancer care in recent years have resulted in increased numbers of people living with and beyond cancer, with increased attention being placed on improving quality of life for those individuals. Connected Health provides the foundations for the transformation of cancer care into a patient-centric model, focused on providing fully connected, personalized support and therapy for the unique needs of each patient. Connected Health creates an opportunity to overcome barriers to health care support among patients diagnosed with chronic conditions. This paper provides an overview of important areas for the foundations of the creation of a new Connected Health paradigm in cancer care. Here we discuss the capabilities of mobile and wearable technologies; we also discuss pervasive and persuasive strategies and device systems to provide multidisciplinary and inclusive approaches for cancer patients for mental well-being, physical activity promotion, and rehabilitation. Several examples already show that there is enthusiasm in strengthening the possibilities offered by Connected Health in persuasive and pervasive technology in cancer care. Developments harnessing the Internet of Things, personalization, patient-centered design, and artificial intelligence help to monitor and assess the health status of cancer patients. Furthermore, this paper analyses the data infrastructure ecosystem for Connected Health and its semantic interoperability with the Connected Health economy ecosystem and its associated barriers. Interoperability is essential when developing Connected Health solutions that integrate with health systems and electronic health records. Given the exponential business growth of the Connected Health economy, there is an urgent need to develop mHealth (mobile health) exponentially, making it both an attractive and challenging market. In conclusion, there is a need for user-centered and multidisciplinary standards of practice to the design, development, evaluation, and implementation of Connected Health interventions in cancer care to ensure their acceptability, practicality, feasibility, effectiveness, affordability, safety, and equity.

## Introduction

Improvements in cancer diagnosis and treatment have resulted in increased survival rates of those suffering from cancer. Some cancer types can now be considered chronic diseases and the attention to patients' quality of life (QoL) during and after cancer treatment is increasing [1]. Consequently, there is an increased burden to the cancer care system due to the increased level of complexity. A clear example of the increased level of treatment complexity in cancer care is the emergence of precision medicine in oncology [2], which relies heavily on the use of biomedical data-driven approaches for choosing the right therapy at the individual and molecular level [3].

According to the World Health Organization, the achievement of universal health coverage to ensure healthy lives and to promote well-being for all is only possible by using new technologies [4]. The advent of Connected Health creates an opportunity to overcome barriers to health care among patients diagnosed with cancer. The technology industry has been developing evidence-based technologies to improve physical activity programs in the clinical setting. The majority of adults in developed countries own mobile phones, therefore, most of the mobile phone-based medical apps aimed at enhancing patient care are easily accessible [5].

Connected Health is defined as follows [6]:

Connected Health encompasses terms such as wireless, digital, electronic, mobile, and telehealth and refers to a conceptual model for health management where devices, services, or interventions are designed around the patient’s needs, and health-related data is shared, in such a way that the patient can receive care in the most proactive and efficient manner possible. All stakeholders in the process are connected by means of timely sharing and presentation of accurate and pertinent information regarding patient status through smarter use of data, devices, communication platforms, and people.

Consequently, Connected Health is about not only technology but also the transformation of health care. Thus, it can provide the foundation for the transformation of cancer care into a patient-centric model focused on providing personalized support to the specific needs of each patient.

In recent years, Connected Health technologies have been applied to cancer care in multiple settings, including screening, patient treatment, after-treatment management, and follow-up of survivors. Connected Health has been promising in managing the quality of care of cancer survivors; this is because it allows the patients to communicate with the health care providers regarding their health status without any dependency on the capacity of a physician [7]. Connected Health allows health care professionals to collect real-time data, which allows medical needs to be identified and responded to within appropriate time frames [7]. Connected Health technologies assist in the evaluation of treatment efficacy by monitoring complications of treatments, such as toxicities or adverse events, and by collecting patient-reported outcome measures (PROMs). Some standard scales and terminologies report these outcomes in a standardized way to be used by digital systems, such as the International Consortium for Health Outcomes Measurement (ICHOM) [8] and the Common Terminology Criteria for Adverse Events (CTCAE) [9]. In addition, new research has shown the importance of PROMs for building integrated cancer care [10]. Limitations to the current application of Connected Health in oncology are the lack of regulation of such technologies and their susceptibility to cybercrime [7].

In this viewpoint paper, we will provide an overview of important areas for the creation of a new Connected Health paradigm in cancer care.

## Cancer Patient Care

### Mental Well-Being

Alongside the physical symptoms associated with cancer and its treatment, people affected by cancer may also experience mental challenges, such as feelings of uncertainty, fear, sadness, or distress [11]. While these symptoms are often transient, at least 15%-20% of cancer survivors will experience disabling levels of distress, such as anxiety or depression, as late as 10 years after their initial diagnosis [12]. Compared to the general population, there is also a greater risk of suicide in cancer survivors [13,14]. Psychological distress can also interfere with one’s cognitive, emotional, and behavioral resources to effectively cope with the impact of living with a diagnosis of cancer [15]. It has been associated with impaired QoL and poor treatment adherence [11]. Psychological distress can also act as a barrier to engaging in recommended long-term self-care activities such as physical activity [16]. Helping cancer survivors better cope with the consequences of cancer and its treatment can enhance their mental well-being, prevent long-term distress, and increase engagement in recommended, positive, health-related behaviors for a better QoL.

Psychosocial interventions, such as cognitive behavioral therapy and behavior change interventions, can enhance coping skills in people affected by cancer [17]. However, face-to-face psychological treatment is costly, often stigmatized, and not always available in hard-to-reach areas. This can result in cancer survivors not having timely access to the psychological support they need [18]. Technological advances in health care and Connected Health, in particular, provide opportunities for increasing access to supportive and individually tailored psychological support for those who need it and can be seen as a first step to seeking support [19]. In supporting mental well-being in people affected by cancer, Connected Health interventions have, to date, focused on the development of Web-based delivery of evidence-based therapies, videoconferencing with health care professionals, and websites for self-care interventions such as coping-skills training [20,21]. While most Connected Health interventions focus on the use of technologies already available and in use in a person’s day-to-day life, gaming technologies and wearables are being adapted for use in supporting mental well-being across age groups [21].

Connected Health interventions for mental well-being in people affected by cancer are generally found to be acceptable; users report high levels of satisfaction with interventions that contain elements of social support, access to health care professionals, and tracking through easy-to-interpret visual representations of measured outcomes [21]. Despite this, the evidence for improvements in psychosocial outcomes remains mixed. While such interventions have been found to be effective in enhancing outcomes such as self-efficacy, coping, and perceived social support, there is limited evidence for their impact on more severe symptoms of distress, such as anxiety or depression [20,21]. These interventions have largely been evaluated in mixed cancer populations, suggesting that they may not cater sufficiently to individual psychosocial needs [22]. Mixed findings can also be explained by the heterogeneity in the technological tools that are used, their exact content, and their duration and intensity of use, as well as in the differences in methods employed for designing, developing, and evaluating these eHealth interventions [23]. While there is promise for the use of eHealth in supporting mental well-being in people affected by cancer, a standardization of practices is needed to ensure that evidence-based technology solutions made available to potential users are effective as well as acceptable, practical, safe, equitable, and affordable [21,23].

Across the development of Connected Health interventions for mental health more broadly, there has been an urgent call for an internationally and cross-disciplinary set of agreed principles and practices for the research and evaluation of digital tools surrounding the intervention’s effectiveness, user experience, and adherence, as well as data safety and privacy [24]. These are equally relevant areas to the design and implementation of Connected Health tools for mental well-being within the cancer population. The effectiveness of an intervention depends upon the correct application of relevant theories and behavior change techniques. However, many Connected Health solutions for mental well-being in people affected by cancer do not provide evidence of this process [21,23,25]. With advances in big data analytics and machine learning algorithms, future developments in this area have the potential to enhance our understanding of behavior change by identifying the digital health strategies that will be most effective in supporting mental well-being based on individual characteristics; this will allow for a more effective and personalized intervention [23]. We need to go beyond effectiveness and promotion of engagement and long-term adherence to the use of such technologies by people affected by cancer; to this end, it is recommended that user-centered design processes, including the involvement of a variety of identified stakeholders and a multidisciplinary team, also be applied from the point of design through evaluation and implementation [23-25].

Advances in technology have enabled new ways to both passively and actively collect user data through methods such as global positioning system, photos, and voice-recording. This highlights the need for guidelines to be developed to ensure potential users have a clear understanding surrounding the storage, use, and sharing of their data [24]. However, this also raises issues with data privacy and safety in the field of mental health care. Torous et al recommend that as technologies advance in this field, there will be a need to undertake technical reviews and audits [24]. Finally, specific regulations and safeguards also need to be implemented to ensure that potential users can objectively assess the quality of Connected Health tools and select the most appropriate tool matching their needs. Evidence-based Connected Health tools for mental health need to be regularly revised and re-evaluated to account for the latest evidence-based knowledge in the area. The application of an evidence-based treatment to Connected Health does not mean that the technology itself is evidence based; it is essential that the technology undergoes its own clinical evaluation [24].

### Physical Activity and Rehabilitation

Physical exercise can be challenging for many patients with cancer due to disease and treatment side effects. Physical rehabilitation in cancer, delivered by health care professionals such as physical therapists and exercise physiologists, assists individuals who have experienced disability in achieving and maintaining optimal functioning in interactions with their environments [26]. Several studies support the integration of physical activity and exercise into ongoing treatment and management across the cancer trajectory [27-29]. Cancer survivors are advised to meet the recommended physical activity levels for adults of at least 150 minutes per week of moderate-to-intense aerobic exercise and muscle-strengthening activities at least two days a week [27,30,31]. A holistic approach that considers disease-related factors, such as severity of the disease, symptoms, and treatment status, and addresses modifiable barriers is essential to facilitate the uptake of physical activity in this population [32-34]. As such, interventions that provide additional support during physical rehabilitation should be considered.

Mobile apps and wearable sensor systems have the potential to support patients and assist in delivering high-quality physical rehabilitation when used as adjuncts to professional treatment. Wearable devices are the most ubiquitous technologies used to track physical activity. As sensors that measure a variety of biological and physiological parameters become smaller and less costly, they can be integrated into wearable systems, which allow for real-time monitoring and evaluation of rehabilitation in a discreet, cohesive manner [35]. When combined with an Internet-enabled interface (eg, a mobile app) and advanced data analytics, these systems can collect and process data and provide personalized feedback to users and to health care providers. Wearable technology is currently used to measure and analyze a wide range of rehabilitation activities and effects; these include movement and physiological responses to exercise as well as health factors closely associated with rehabilitation, such as sleep and general physical activity [35,36]. These devices provide real-time feedback and allow health care professionals to remotely monitor clinically relevant variables in a nonclinical setting. While there are opportunities for consumer-grade wearable systems in oncology practice [37], in comparison to bespoke rehabilitation systems, these lack clinical validity and are not equipped to conduct human motion analysis at the level often required in a rehabilitation setting [38,39].

Wearable technologies can be used to deliver exercise to those who may experience exercise-limiting symptoms. Wearable neuromuscular electrical stimulation (NMES) technology may be a pragmatic alternative to voluntary aerobic and resistance exercise in such cases. NMES involves the contraction of skeletal muscles via electrical impulses delivered to motor nerves using surface electrodes placed over target muscle groups [40]. Traditional high-frequency NMES (20-100Hz) has been extensively used in sports training and in different rehabilitation contexts to augment muscle mass and strength [41]. In addition, emerging evidence demonstrates the efficacy of low-frequency NMES (3-12 Hz) for enhancing cardiorespiratory fitness [42]. Furthermore, wearable NMES technology allows for the delivery of NMES exercise sessions safely and unsupervised in the user’s home; this makes it an attractive technology for patients with cancer who may be physically deconditioned, with impaired cardiorespiratory and muscular fitness. Previous work has attempted to implement NMES exercise into the rehabilitation of advanced cancer patients. The NMES exercise interventions used were adapted from orthopedic and neurological rehabilitation contexts and were largely unsuccessful due to inappropriately designed protocols [43,44]. Recent work using a personalized and progressive NMES exercise approach, designed with early-stage cancer rehabilitation in mind, has reported improvements in functional strength and QoL outcomes [45]. This preliminary evidence highlights the potential of wearable systems such as NMES exercise technology in cancer rehabilitation.

Providing patients with additional support during exercising may help reduce barriers to home-based rehabilitation. One such digital rehabilitation technology is biofeedback, which is the process of providing an individual with information regarding a particular body function and allows the individual to self-regulate this function. Through visual, audio, haptic, or multi-model feedback delivered in real time or after exercising, patients receive personalized guidance on their rehabilitation. This may help enhance motor learning and improve engagement with therapy [46,47]. Biofeedback systems in cancer rehabilitation should be codesigned with users for specific clinical contexts to ensure that they are meaningful, accessible, and clinically effective [48,49]. Including appropriate elements of gamification (eg, points and levels) in the software development process may help promote engagement and adherence in some cohorts [50,51].

Gamification helps make everyday tasks more interesting and, thus, potentially more engaging for the users [52]. The health care industry continuously embraces this idea of gamification as a way of encouraging patients and individuals diagnosed with chronic diseases to enjoy physical activities. Gamification refers to the process of using the principles found in game design in a nongame context. Studies reveal that gamification technology in health care is the best way of engaging people in physical activities [53]. However, it is important to understand that the patient’s motivation to engage in physical activities through mobile technology may be influenced by social interaction, attitude, and individual personality [53,54]. Therefore, health care practitioners may need to consider goal-oriented activities that will help patients understand the primary objectives of the games on the mobile technology along with aspects that will influence patients’ engagement with the games.

Promoting physical activity among cancer survivors remains a significant priority in health care. Health care practitioners are continuously looking for ways they can enhance patient engagement in physical activities [55]. The introduction of Connected Health presents limitless possibilities because it strengthens the patient-practitioner relationship. Connected Health allows coaching of physical activity because it enables exchange of valuable information between the patient and health care practitioner that can help in managing the patient’s physical activity [56]. In a recent study, a continuous wrist-mounted tracker monitored physical activity with daily step counts in 38 patients with different cancer types undergoing chemoradiotherapy with curative intent; physical activity monitored by the tracker correlated significantly with the risk of unplanned hospitalization during or shortly after therapy, which suggested a role for this technology as a dynamic indicator of this potentially life-threatening adverse event [57].

Additionally, clinicians may benefit from having quantifiable, reliable, and objective measures of therapeutic assessments and interventions that were previously not possible [58]. They may also benefit from high-volume, longitudinal, granular data, with which they can analyze disease management and demonstrate outcomes to commissioners. Researchers using these systems will gain the ability to collect large-scale, momentary, longitudinal data; quantify intervention outcomes; and supplement existing QoL assessment tools.

## Persuasive and Pervasive Technology in Cancer Care

### Internet of Things

The use of pervasive computing solutions, such as mobile phones, wearables, or connected home appliances, can help monitor and assess the health status of patients. These environments of connected devices can be described within the concept of *Internet of Things*, which was defined by Gartner as “the network of physical objects that contain embedded technology to communicate and sense or interact with their internal states or the external environment” [59]. These connected technologies provide multiple ways to monitor the real-time health status of patients by sensing the individual and his or her environment [60]. More advanced systems that can be worn by patients include textile wearable devices; flexible, stretchable, and printable devices (eg, epidermal electronics and e-skin); and devices embedded into a patient's living environment.

It has been shown that even the simplest pervasive technology, such as regular phone calls and mobile phone apps, used for structured follow-up intervention provides improvements in pain, depression, distress, and QoL. These studies were focused on patients after completion of their primary treatment of breast, prostate, colorectal, and lung cancer [61].

For purposes of cancer care, sensing systems can be used for objective older adult patient monitoring for baseline functional status and treatment toxicity [62]. For immobile patients, the big challenge is prevention of pressure ulcers. Wearable technology can support health care providers in keeping turning schedules for large groups of patients [63]. Skin conductance or temperature can provide important insights into physiologic changes in cancer patients. Textile wearable technology integrates sensor, energy sources, processing, and communication devices within the garment. An example of such a system is a bra that helps to detect breast cancer using intelligent breast patches that detect small circadian temperature changes in breast cells and communicate this via mobile phone to a designated remote health center [64]. Electronic circuits in flexible electronics are manufactured or printed on flexible substrates (eg, cloth fabrics and the human body). It can be used to detect specific biomarkers, such as complement proteins associated with breast cancer from saliva, tears, or the breath [65]. Epidermal electronics, with their stretchability, enable devices to bend to a very small radius and still maintain the desired electronic performance. This allows the design of an ultra-thin surface electromyography patch for swallowing-therapy exercises for head and neck cancer patients [66]. The photoplethysmographic sensing method enables assessment of cuffless blood pressure using the integration of electrodes into chair pads and arms or into beds under the mattress [67].

A cognitive perspective presented in Lucchiarri et al [68] is related to methods for nutrition monitoring in cancer patients. Inspiration for food classification and estimation (eg, macronutrient content) can be found from other chronic diseases like diabetes [69]. The solution presented in this paper was developed by a research team at the National Technical University of Athens and is based on the classification system for food images taken by a mobile phone camera and exploiting convolutional neural networks.

### Personalization

An underlying challenge of mobile health (mHealth) apps is user abandonment [70-72]. This problem has also been reported in studies with cancer survivors, with the majority of users ceasing app usage within 1-3 months [73,74]. Among the factors associated with this lack of long-term engagement is the feeling of low perceived personal relevance in the experience provided by these apps. It is believed that mobile-based interventions that are closely tailored to the individual’s convictions and motivations are more likely to be observed and remembered [75]. Therefore, personalization or tailoring can help increase the intended effects of communication, which can contribute to overcoming user abandonment and improving the effectiveness of these systems [76]. This need for tailored experiences has been highlighted in several studies with cancer survivors [77-79]. mHealth apps can address this need as they provide an opportunity to access actionable individual-level data; this facilitates the tailoring of interventions to users’ personal information, such as treatment history, age, gender, stage of change, fitness level, cognitive flexibility, or health goals [74]. However, there is a paucity of research exploring this topic. Only recently has there been research on mHealth tools to connect patients with timely and actionable educational health information [78]. Overall, mHealth systems for cancer lack the personalization that could engage users in their long-term adoption.

Cancer survivors reported a preference for an app that is highly individualized to them and suggested that the app should offer the following: content that is sensitive to user-identified information (eg, cancer diagnosis, personal health considerations, age, and physical limitations); a feature to self-monitor changes in health indicators (eg, heart rate, cholesterol, and waist measurement); personalized goal suggestions; personalized role model narratives; suggestions based on users’ location and weather; and adaptation to trends over time [79].

The theory and practice in the field of computer-tailored health communication [80-84], particularly the one applied to real-time coaching systems [85], proposes a variety of approaches to adapt the four different properties of communication—timing, intention, content, and representation—to create more individual technological experiences. These approaches include the following:

1. Feedback: presenting the user with information about themselves.
2. Interhuman interaction: support for interaction with other people with a similar condition.
3. Adaptation: direct messages regarding an individual’s status on key theoretical determinants.
4. User targeting: increase attention or motivation by conveying that the communication is designed specifically for the user.
5. Goal setting: learn user-specific goals based on individual patterns.
6. Context awareness: tailoring communication based on external information.
7. Self-learning: learning about the user’s reactions to previous communications.

Such strategies are aligned with the tailoring needs reported by cancer survivors [77,79,80] and can be used to complement the guidelines for empowering cancer survivors through mobile apps [86,87].

A recent randomized controlled trial tested a technology-enhanced lifestyle program in prostate cancer patients after treatment for localized disease. Prostate cancer patients are at risk of relapse and lifestyle behaviors may reduce this risk. A combination of a website, Fitbit One, and short message service (SMS) text messaging were used to facilitate the adoption of eight behaviors, including vigorous activity, smoking cessation, and dietary improvements. As compared with the control group (32 participants in each arm), significantly more patients in the intervention group modified the score of lifestyle behaviors, mainly driven by diet rather than exercise [88]. This demonstrates feasibility and a high degree of patient acceptability of these interventions that combine education, longitudinal collection of data, and some interface with the Internet or connected devices.

Even though the potential for personalization in mHealth systems seems clear, there are still many barriers that need to be overcome. These include frequent manual input from the user, which can be perceived as burdensome and may decrease interest in app use. Real-time monitoring technologies can partly reduce this entry burden. However, obtaining monitoring data for timely coaching is not as of yet a straightforward process. Most commercially available activity-monitoring devices only allow the capture of data through their Web application programming interfaces, which results in significant delays from the moment the data is collected until the feedback can be provided to the user. An alternative is to collect the raw data from the sensors, which can be done either from these external wearable devices with the appropriate permissions or from the built-in sensors in the device. However, this normally requires complex processing to make sense of such data and involves extra considerations in terms of hardware and software compatibilities.

Currently, there is still little involvement of cancer health care stakeholders in the design and development of these apps. When it comes to mHealth system evaluation, adopting the optimal methodological approaches may be time and resource consuming and, therefore, challenging to put into practice. Hence, there is a paucity of studies evaluating these systems in a structured and controlled manner and assessing the long-term effects [73]. Furthermore, there is a lack of proper evaluation of particular persuasive mechanisms such as those related to providing a personalized experience on user engagement and behavior change, which introduces a challenge regarding the choice of particular strategies for the design of these systems [73].

The integration with electronic health records (EHRs) may significantly help create personalized information for the users; however, this may also be used to generate meaningful health data that can be shared with the professionals. This will reduce manual data entry by users and will increase the value, reliability, and credibility in these systems [89]. Furthermore, developments in mobile and wearable technologies will continue increasing the capability of mHealth apps to monitor health parameters and will bring new opportunities for real-time feedback and motivation. New and more intelligent forms of personalization will be implemented, which can potentially make a difference in maintaining user engagement and increase the intended effects of these systems. Systems will, for example, be able to dynamically adapt content and functionality according to patients’ most pressing needs. The *user* can be seen as a dynamic entity; apps will be required to update their internal model of the user by recording and learning from the user’s interactions with the app [85]. These apps will then be able to make sense of the user’s schedule and routine, progress, and preferences for certain features or particular information. In addition, this information may be used to help create an app experience that is more targeted to the individual needs of the patient.

Personalized mHealth apps can potentially benefit cancer survivors by providing a platform for self-tracking and self-management. This may help gather information on their condition, which may enhance communication with health care professionals and increase self-awareness and self-confidence in achieving health goals. With the rapid rise of mobile phone use and the increase in the complexity and accuracy of mobile monitoring technologies, these apps can now gather large amounts of users’ health data and provide information and motivation anytime and anywhere to these individuals. Future research is needed that incorporates theory and practice of computer-tailored health communication in these mHealth systems; this would help leverage individual data and provide highly personalized support and encouragement for self-management in cancer survivorship.

### Artificial Intelligence and Machine Learning

The use of digital tools to gather health-related data from patients is widely accepted. However, examining how this data can be applied to enhance and adapt the provision of care to meet individual patients’ needs across the health care pathway is still in its early stages. Artificial Intelligence (AI) techniques, based on directly and indirectly reported patient data, have the potential to create personalized supportive recommendations. While the future of AI in cancer care is promising, further research is needed before this type of data-driven solution can become an established method for enhancing patient care.

In the provision of cancer care, the application of AI has been explored as a means to support an earlier and more accurate diagnosis and to improve prognosis [90]. AI aims to go beyond evidence-based medicine by extending the validated and reported knowledge acquired through the discovery of new insights; this will be done by applying data-driven approaches. Hence, it can extend the knowledge derived from model-driven approaches, such as clinical practice guidelines, by providing new patterns and understanding discovered through the analysis of patients’ health-related data. One of the most popular applications of AI is machine learning, which has been used to predict cancer patients’ responses to different drug treatments. For example, a recent study [91] showed that AI can predict treatment response, with more than 80% accuracy, by combining learning techniques with extensive patient data. That prediction may help to provide patients with the most effective treatment for them, thereby improving their experience through their patient journey as well as their health outcomes.

However, AI can go beyond enhancing clinical decision support. For instance, a 2018 study evaluated the impact of using AI to optimize the management of cancer-related pain. Through a randomized controlled trial, it was found that AI reduced perceived pain in 20% of patients and decreased inpatient hospital admissions by 40%. Although this solution increased anxiety, it shows that AI has the potential to be applied in more innovative ways within the cancer care pathway [92]. AI can also be used to investigate and analyze cancer patients’ behaviors and emotions to generate actionable insights that have the potential to improve patient health-related outcomes. For example, there exists a ready-to-use solution—the recently presented Patient-Reported Information Multidimensional Exploration (PRIME) framework [93]—which uses machine learning and natural language processing on the contents of online patient support groups. This framework is applicable to different cancer types and its code is available online for use [94].

Furthermore, cancer patients’ health-related behaviors, such as maintaining a physically active lifestyle, can alleviate cancer-related symptoms including fatigue. AI has previously been applied in other areas of health to influence patients’ health-related behaviors, for instance, healthy eating [95] or smoking cessation [96]. However, few of these applications combine AI with validated models of behavior change. Hence, by taking into consideration nuances of cancer patients’ needs in combination with behavioral change models, it is possible to create more robust solutions.

## The Data Infrastructure Ecosystem

Cooperative care requires operability between all principals in health care, including persons, organizations, devices, applications, and components [97]. The European Commission has officially recognized the need for improving interoperability in Connected Health and has allocated resources to this end. For example, the 2018 eHealth Interoperability Conformity Assessment Scheme for Europe (EURO-CAS) is paving the way for more eHealth interoperability in Europe [98]. This shows evidence for the need for processes and protocols to enable interoperability. The researchers, health care professionals, and other involved parties in the health care ecosystem are well aware of these needs. As such, several organizations for developing standards for health care interoperability have been created, such as Health Level Seven (HL7) International, the International Health Terminology Standards Development Organisation (IHTSDO), the Organization for the Advancement of Structured Information Standards (OASIS), and the Object Management Group (OMG) [97]. They have proposed several approaches for increasing Connected Health interoperability, with initiatives such as Fast Healthcare Interoperability Resources (FHIR), perhaps the most promising up to now for its easy implementation.

Interoperability is key when developing Connected Health solutions because it highly affects integration with health systems and the EHR system [99], thus making these solutions more useful. FHIR came from HL7 [100], which presented shortcomings in the form of needs for complex custom tooling. FHIR aimed to simplify and accelerate HL7 adoption by being easily consumable but robust and by using open Internet standards, where possible, using an easily consumable format. The FHIR approach was based on the representational state transfer (RESTful) principles described by Fielding [101]. FHIR is considered the de facto standard for interoperability in health recommender systems and many solutions are built on top of that [102,103].

The field of PROMs, adverse events, and QoL is of great interest for data collection and further intervention in cancer care. Digital platforms may facilitate self-reporting of signs and symptoms that correlate with other relevant outcomes, such as performance status, treatment adherence, and survival. Data capture in real time of validated measures of PROMs and QoL is facilitated by wearable devices, which have been shown to be equivalent to previous techniques [104]. Interestingly, Web-based symptom monitoring during chemotherapy among breast, genitourinary, gynecologic, and lung cancer patients was associated with improved QoL, fewer visits to the emergency room, and fewer hospitalizations than among the nonintervention group [105]. Furthermore, in a subsequent analysis of overall survival, there was a 5-month, statistically significant difference in survival favoring the intervention group [106].

## The Connected Health Economy Ecosystem

Health care systems worldwide currently struggle with challenges of an aging population alongside budgetary pressures. Connected Health is an emerging field with the potential to be a relevant driver in the needed transformation of health care for increasing its quality and efficiency. mHealth allows the implementation of tools to tackle these challenges by improving the efficiency of the health care system and supporting the shift toward prevention [4].

Sensors and mobile apps gather considerable amounts of medical, physiological, lifestyle, daily activity, and environmental data, which could serve as a basis for evidence-driven practice and research activities. mHealth also facilitates patients’ access to their data anytime and anywhere and, therefore, facilitates patient empowerment. Hence, it also enhances the connection between the patient and health care professional, thus making it more efficient across contexts. For example, with the help of self-assessment or remote monitoring solutions, patients could manage to live more independently in their home environment. With this additional data, the health care professional may be able to make a more accurate assessment and provide better personalized treatments. In addition, mobile apps can also be used to encourage adherence to a healthy lifestyle.

The worldwide uptake of Connected Health can be linked to the growth in wireless subscriptions, which has reached over 6 billion internationally [107]. The fast development of communication technologies and health care devices, alongside the social demand, are creating new businesses focusing within highly promising markets. Market potential differs depending on the context of each individual country, thus shaping business. While high-income countries are driven by cutting health care costs and giving patients a more active role in their own health care, developing countries are focusing on improving access to primary care [4].

Approximately 70% of mHealth apps target the consumer wellness and fitness segments. The other 30% target health care professionals by facilitating access to patient data, patient consultation, monitoring, diagnostic imaging, and access to pharmaceutical information, among others [108]. The European Union estimated that there were 97,000 mHealth apps and 3 billion mHealth app downloads in 2015 [109], with these numbers continuing to grow. However, the global market report on mHealth mentions that almost 70% of these apps did not reach sales of US $10,000 per year [109]. The lack of success in monetization of mHealth apps reveals how difficult it is to reach sustainability in this sector. Nevertheless, mHealth business is growing exponentially, from US $5.7 billion in 2015 to an estimation of US $31 billion in 2020, making it both an attractive and challenging market [109].

Digital solutions facilitate complex monetization models where different and complementary models can coexist in a single app, making the market appealing and challenging at the same time. Monetization models can be classified into eight groups:

1. Free: there is no payment for the use of the app.
2. Download: one payment for a license for use.
3. Subscription: a license for a period of time.
4. Platform: income for installation and use in an organization.
5. Service: income for a service, content, and products.
6. Marketing: incomes for advertisements and content placement.
7. Results based: the improvement of the efficiency and/or health of patients.
8. Assets: income based on selling data generated by the use of the app.

In addition, it is possible to have multiple monetization models for a single solution. One app can be used by different stakeholders at the same time in different ways with different needs, which means complementary monetization paths; for example, free for the user but using marketing (eg, advertisements or contents) unless the user pays a premium service.

One of the most challenging monetization models is the building of assets based on data generated by users. mHealth apps can gather large quantities of information, which can be processed for different purposes. Big data analysis is the capacity to analyze large amounts of unstructured datasets from a wide range of sources, involving the extraction of potentially valuable information in a cost-effective way. Therefore, the use of health-related data is a central element of epidemiological research as it can enable the identification of patterns to improve treatments, optimize trial periods for medication, and advance mechanisms for early detection and prevention of diseases. This potential also allows for the development of new, innovative business models in health care.

## Connected Health Barriers

The popularization of mobile phones and, more recently, wearable devices is creating a large volume of population data and creates an opportunity for Connected Health in cancer care. It is likely that future patients will generate their own data, either because such devices have become part of the care practice model or because these devices are part of the patients’ lives. Consequently, data interoperability problems may become even more relevant than they are today. For instance, one of the most popular wearable devices for tracking health-related information that may contribute to developing AI-based solutions for cancer patients is the Fitbit, which still uses a proprietary architecture. This hampers its usage in AI systems that are compatible with data following existing industry standards, such as HL7 Consolidated Clinical Document Architecture (C-CDA) or the upcoming FHIR.

Nevertheless, the adoption of Connected Health in cancer care still has many barriers to address. These systems need a robust regulatory framework to ensure they are of high quality, they demonstrate clinical effectiveness, and that privacy and security are respected and protected. Existing limitations are related to an inherent digital divide, concerns about privacy, data volume, cost, and reimbursement. It is imperative that these solutions are interoperable and have the potential to be smoothly synchronized with EHRs. Indeed, if siloed, the capacity for Connected Health solutions will be limited regarding their support for cancer patients as part of the digital-based cancer care system to which developing countries are transitioning. In this context, there already exists standardized communication frameworks and data architectures to facilitate such interoperability in the data provided by Connected Health cancer solutions.

Regardless of the data origin, the reasoning behind the recommendations generated by AI-based systems often remains difficult to explain, as these systems can be opaque and nonintuitive. This may result in a lack of trust in AI-based systems, reducing their adoption by patients, and may also prevent health care professionals from prescribing them. Future systems will have to strive to explain how recommendations have been computed [110].

## Privacy and Data Protection

The processing of data concerning personal health records is particularly sensitive and requires special protection. Personal data protection is a fundamental right in Europe and, therefore, vital for building trust in mHealth solutions [111].

This is particularly important considering these personalization technologies may deal with sensitive personal data from users. Even if technologies adopt the appropriate information technology infrastructure for privacy requirements, users can be hesitant to share their personal information. Patients need to think about the trade-off between maintaining privacy of the data collected through these mHealth apps and the potential benefit this might bring in facilitating a more individualized and engaging experience [112]. Patients also need to feel that they can rely on the app; they should also be aware that the information is coming from a trustworthy source (eg, their cancer care providers or an authoritative health agency) or that experts and health care professionals have validated it.

Connected Health solutions should be especially concerned with ensuring data protection and privacy for all individual citizens of the European Union and the European Economic Area. Moreover, given the international component of connected services, addressing the transfer of personal data outside the European Union and the European Economic Area would be of high importance. This may be in compliance of the General Data Protection Regulation [113] by giving control to individuals and by homogenizing the environment for international business activities relative to personal data.

## Conclusions

Connected Health in cancer care aims to reduce barriers to health care provision through the development of well-designed, usable, and validated digital solutions. There is an urgent need for user-centered and theory-driven international and multidisciplinary standards of practice to inform the design, development, evaluation, and implementation of Connected Health interventions in cancer care. This can ensure that solutions are seen as acceptable, practical, effective, affordable, safe, and equitable. Including users in the design process, testing extensively, and committing to the development of a regulatory framework for software components are strategies that need to be adopted when developing a Connected Health app for cancer care.

Regarding the technology developments currently available in Connected Health, there is early evidence showing that AI can be used to compute recommendations aiming to support cancer patients. Classical model approaches can then be augmented with the new evidence inferred from data analytics. Increasing sources of patient data can enhance the potential of AI by facilitating opportunities for improved user modeling and evaluation of the actual evidence through this data. The Connected Health market is growing and there are many ways it can be made economically viable. The volume of generated data offers great opportunities to extract actionable information for patients; however, interoperable and comprehensive approaches to their use are crucial. The field of machine learning and advances in big data analysis, in particular, provide opportunities for the development of effective Connected Health interventions in cancer by identifying the content and extent of support needed by a user: that is, being able to identify what intervention works for whom and under what circumstances.

## Abbreviations

AI: artificial intelligence
CATCH: Cancer: Activating Technology for Connected Health
C-CDA: Consolidated Clinical Document Architecture
COST: European Cooperation in Science and Technology
CTCAE: Common Terminology Criteria for Adverse Events
EHR: electronic health record
ENJECT: European Network for the Joint Evaluation of Connected Health Technologies
EURO-CAS: eHealth Interoperability Conformity Assessment Scheme for Europe
FCT: Fundação para a Ciência e a Tecnologia
FHIR: Fast Healthcare Interoperability Resources
HL7: Health Level Seven
ICHOM: International Consortium for Health Outcomes Measurement
IHTSDO: International Health Terminology Standards Development Organisation
mHealth: mobile health
NMES: neuromuscular electrical stimulation
OASIS: Organization for the Advancement of Structured Information Standards
OMG: Object Management Group
PRIME: Patient-Reported Information Multidimensional Exploration
PROM: patient-reported outcome measure
QoL: quality of life
RESTful: representational state transfer
SMS: short message service
